# Supplementary material for: Molecular mechanism of ultrafast transport by plasma membrane Ca2+-ATPases
Source: Nature. 2025 Aug 20;646(8083):236–45. doi: 10.1038/s41586-025-09402-3 (PMC12488499; doi:10.1038/s41586-025-09402-3)
Supplement: Supplementary file 1 — This file contains Supplementary Figures 1–5 and Supplementary Tables 1–4. [file 41586_2025_9402_MOESM1_ESM.pdf]

nature portfolio

https://doi.org/10.1038/s41586-025-09402-3

Supplementary information

Molecular mechanism of ultrafast transport by plasma membrane Ca^{2+}-ATPases

In the format provided by the authors and unedited

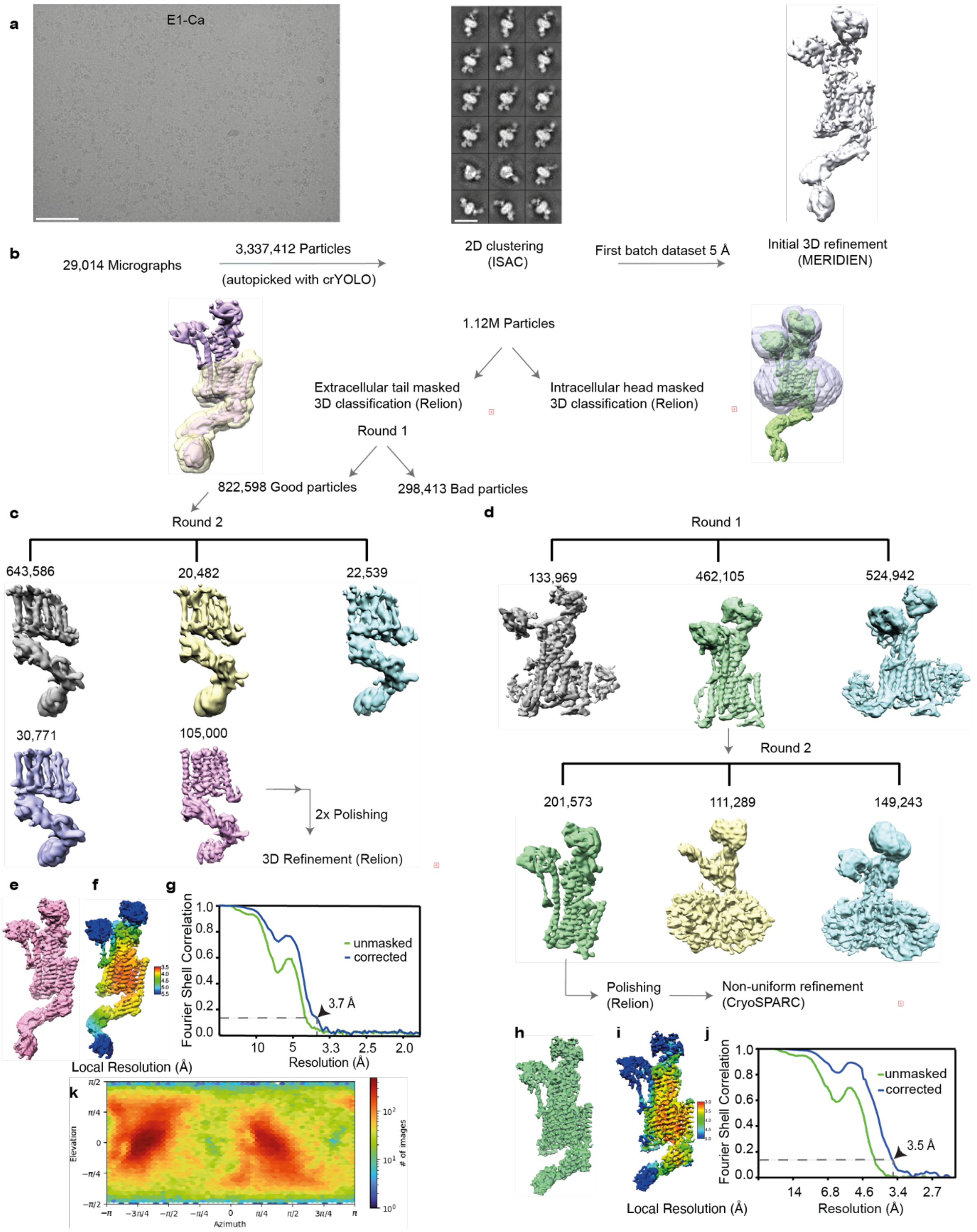

Supplementary Figure 1. Cryo-EM image processing workflow for the PMCA2-NPTN complex in the E1-Ca state. a, Representative digital micrograph image and selected 2D class averages of the PMCA2-NPTN complex in the E1-Ca state. The initial refinement density obtained with MERIDIEN is shown next to the 2D class averages. Scale bars: 50 nm and 10 nm, respectively. **b, c, d,** The masked 3D classification workflow using either an extracellular tail mask **(c)** or an intracellular head mask (**d**). **e, h,** Final maps obtained after classification and 3D refinement. **f,i,** Local resolution estimations of the maps, calculated by CryoSPARC. **g,j,** Fourier shell correlation curves (FSC) between the two independently refined particle subsets. The dotted lines indicate the FSC=0.143 criterion used for average resolution estimation. **k**, Angular distribution of the particles used for the reconstruction of the final E1-Ca map in (**h**).

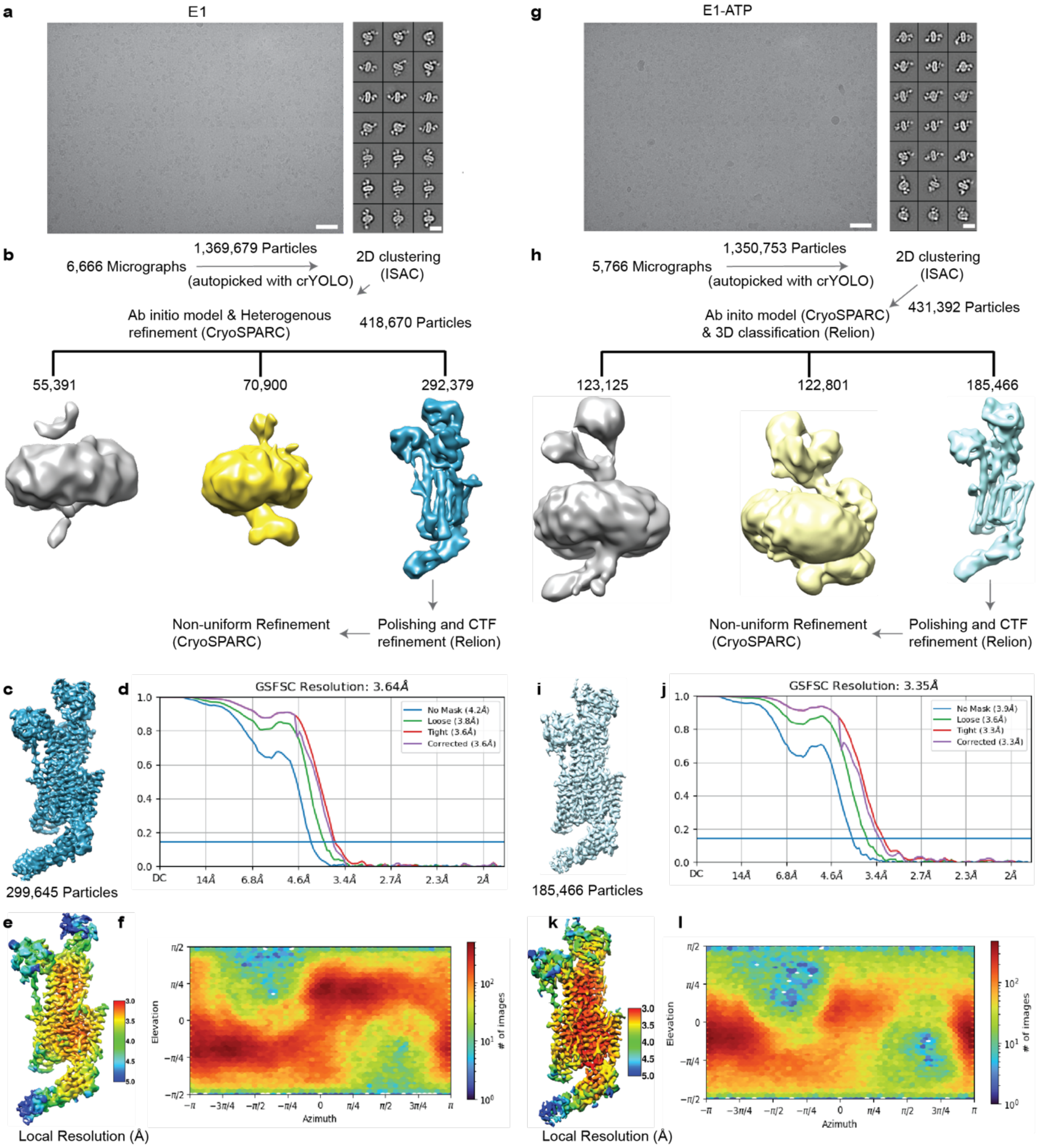

Supplementary Figure 2. Cryo-EM image processing workflow for the PMCA2-NPTN complex in the E1 and E1-ATP state. a, Representative digital micrograph and selected 2D class averages of the PMCA2-NPTN complex in the E1 state. Scale bars: 50 nm and 10 nm, respectively. **b**, Workflow used to obtain the high-resolution structure of the E1 state. **c,d,** Final high-resolution map and the FSC corresponding to two independently refined particle subsets for the E1 state. **e,** Local resolution estimations of the E1 density map, calculated by CryoSPARC. The bar depicts local resolution in Å. **f,** Angular distribution of the particles used for the reconstruction of the final E1 map. **g-l,** as (**b-f**) but for the E1-ATP state of the PMCA2-NPTN complex.

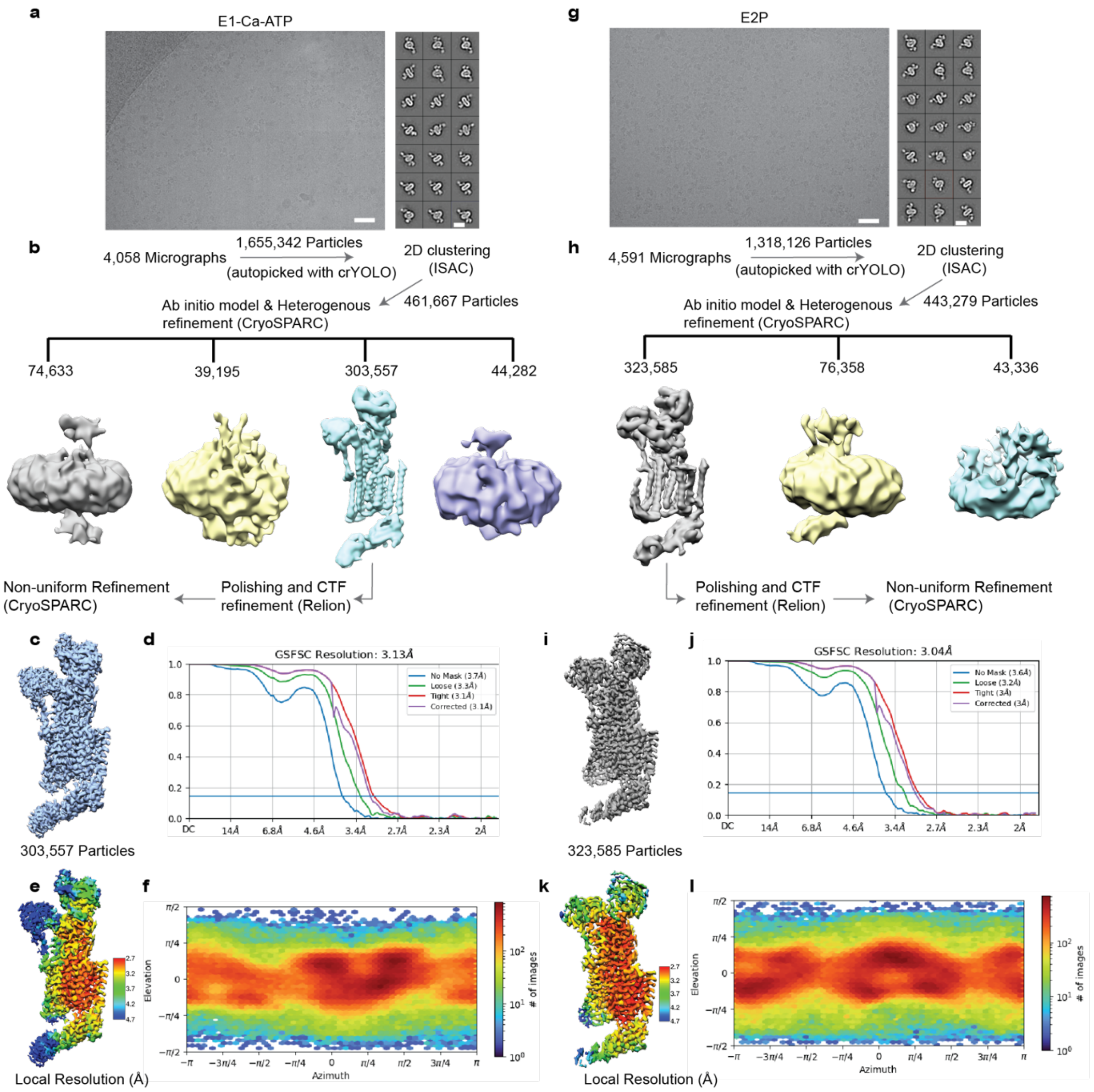

Supplementary Figure 3. Cryo-EM image processing workflow for the PMCA2-NPTN complex in the E1-Ca-ATP and E2 state. a, Representative digital micrograph and selected 2D class averages of the PMCA2-NPTN complex in the E1-Ca-ATP state. Scale bars: 50 nm and 10 nm, respectively. **b**, Workflow used to obtain the high-resolution structure of the E1-Ca-ATP state. **c,d,** Final high-resolution map and the FSC corresponding to two independently refined particle subsets for the E1-Ca-ATP state. **e,** Local resolution estimations of the E1-Ca-ATP density map, calculated by CryoSPARC. The bar depicts local resolution in Å. **f**, Angular distribution of the particles used for the reconstruction of the final E1-Ca-ATP map. **g-l,** as (**b-f**) but for the E2P state of the PMCA2-NPTN complex.

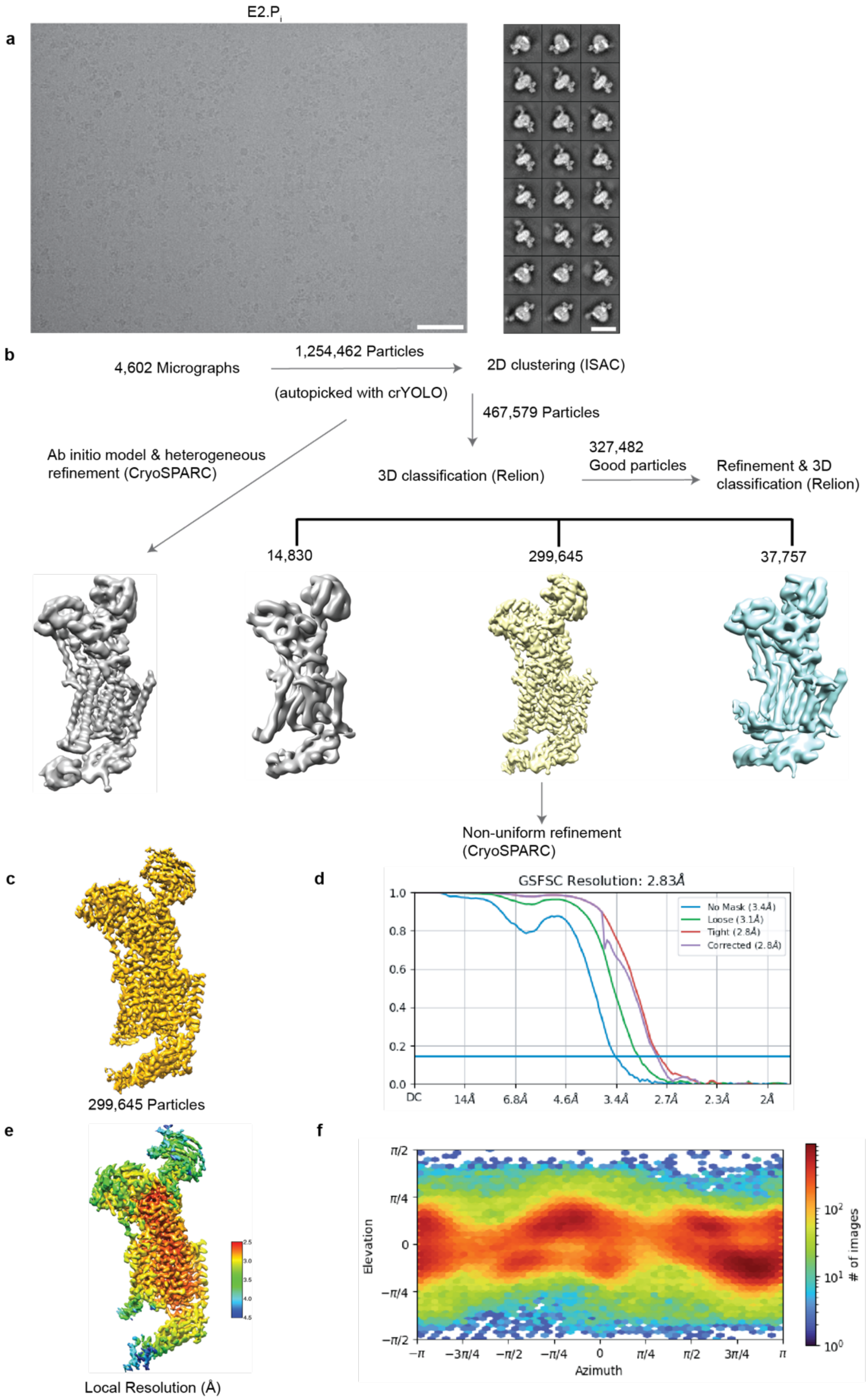

Supplementary Figure 4. Cryo-EM image processing workflow for the PMCA2-NPTN complex in the of E2.P$_i$ state. a, Representative digital micrograph and selected 2D class averages of the PMCA2-NPTN complex in the E2.P$_i$ state. Scale bars: 50 nm and 10 nm, respectively. **b**, The initial density obtained after *ab initio* model building and heterogeneous refinement and the workflow used to obtain the high-resolution structure of the E2-P$_i$ state. **c,d,** Final high-resolution map and the FSC corresponding to two independently refined particle subsets for the E2-P$_i$ state. **e,** Local resolution estimations of the E2.P$_i$ density map, calculated by CryoSPARC. The bar depicts local resolution in Å. **f**, Angular distribution of the particles used for the reconstruction of the final E2-P$_i$ map.

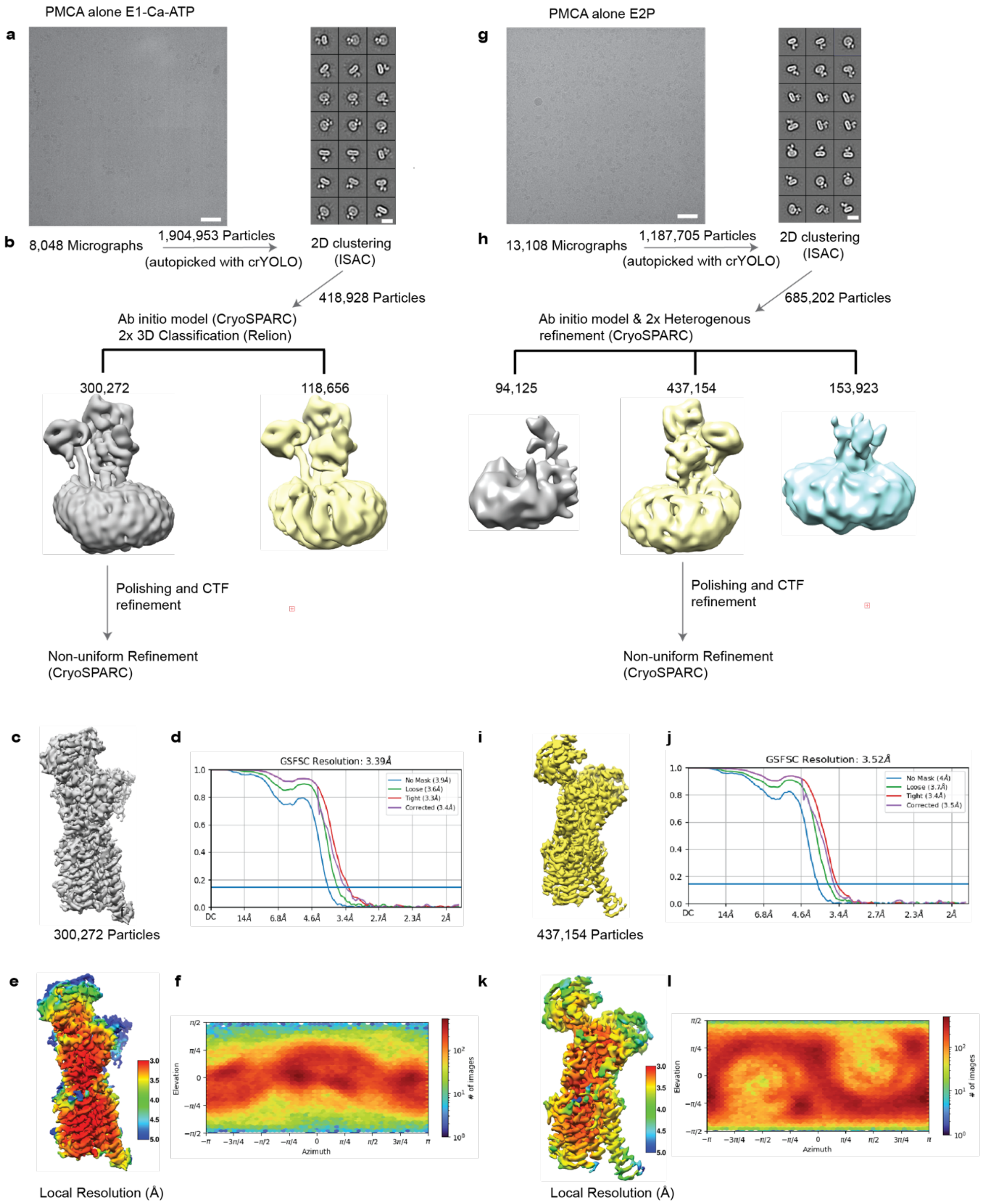

Supplementary Figure 5. Cryo-EM image processing workflow for the PMCA2 in the E1-Ca-ATP and the E2P state. a, Representative digital micrograph and selected 2D class averages of PMCA2 in the E1-Ca-ATP state. Scale bars: 50 nm and 10 nm, respectively. **b**, Workflow used to obtain the high-resolution structure of the E1-Ca-ATP state. **c,d,** Final high-resolution map and the FSC corresponding to two independently refined particle subsets for the E1-Ca-ATP state. **e,** Local resolution estimations of the E1-Ca-ATP density map, calculated by CryoSPARC. The bar depicts local resolution in Å. **f**, Angular distribution of the particles used for the reconstruction of the final E1-Ca-ATP map. **g-l,** as (**b-f**) but for the E2P state of PMCA2.

Structure	Solubilization	Washing 1 (WP1)	Washing 2 (WP2)
PMCA2-NPTN<br>E1-Ca	ComplexioLyte 145<br>0.5 mM EDTA+EGTA<br>later: 35 mM imidazole	50 mM imidazole<br>0.1 % GDN	0.1 % GDN
PMCA2-NPTN<br>E1-Ca-ATP	ComplexioLyte 145<br>0.5 mM EDTA+EGTA<br>later: 30 mM imidazole<br>2.5 mM $MgCl_2$<br>1 mM ATP	40 mM imidazole<br>1 mM MgATP<br>0.05 % GDN	0.025 % GDN<br>2 mM AMPPNP<br>2 mM $MgCl_2$
PMCA2-NPTN<br>E1	ComplexioLyte 145<br>0.5 mM EDTA+EGTA<br>later: 30 mM imidazole<br>2.5 mM $MgCl_2$<br>1 mM ATP	40 mM imidazole<br>1 mM MgATP<br>0.05 % GDN	0.025 % GDN<br>10 mM EGTA
PMCA2-NPTN<br>E1-ATP	ComplexioLyte 145<br>0.5 mM EDTA+EGTA<br>later: 30 mM imidazole<br>2.5 mM $MgCl_2$<br>1 mM ATP	40 mM imidazole<br>1 mM MgATP<br>0.05 % GDN	0.025 % GDN<br>2 mM AMPPNP<br>2 mM $MgCl_2$<br>10 mM EGTA
PMCA2-NPTN<br>E2.P_i	ComplexioLyte 145<br>0.5 mM EDTA+EGTA<br>later: 30 mM imidazole<br>2.5 mM $MgCl_2$<br>1 mM ATP	40 mM imidazole<br>1 mM MgATP<br>0.05 % GDN	0.025 % GDN<br>3.2 mM $NaAlF_4$ +<br>0.4 mM ADP (+ 4<br>mM MgADP in the<br>elution buffer)
PMCA2-NPTN<br>E2P	ComplexioLyte 145<br>0.5 mM EDTA+EGTA<br>later: 30 mM imidazole<br>2.5 mM $MgCl_2$<br>1 mM ATP	40 mM imidazole<br>1 mM MgATP<br>0.05 % GDN	0.025 % GDN<br>5 mM NaF + 0.6<br>mM BeF_3
PMCA2<br>E1-Ca-ATP	ComplexioLyte 145<br>0.5 mM EDTA+EGTA<br>later: 15 mM imidazole<br>2.5 mM $MgCl_2$<br>1 mM ATP	20 mM imidazole<br>1 mM MgATP<br>0.05 % GDN	0.02 % GDN<br>2 mM AMPPNP<br>2 mM $MgCl_2$
PMCA2<br>E2P	ComplexioLyte 145<br>0.5 mM EDTA+EGTA<br>later: 15 mM imidazole<br>2.5 mM $MgCl_2$<br>1 mM ATP	20 mM imidazole<br>1 mM MgATP<br>0.05 % GDN	0.015 % GDN<br>5 mM NaF + 0.6<br>mM BeF_3

Supplementary Table 1. PMCA2 protein sample preparation summary

Protein	Domain	Movement	Transition E1 to E1-Ca	Transition E1-Ca to E1-Ca-ATP	Transition E1-Ca-ATP to E2P	Transition E2P to E2.Pi	Transition E2.Pi to E1
**PMCA2**	A	Shift (Å)	3,2	0,1	-4,6	1,4	-3,7
**SERCA1A**	A	Shift (Å)	3,9	3,6	-5,3	3,2	-5,0
**SERCA2B**	A	Shift (Å)	-	-0,4	-6,2	4,0	-
**SPCA1**	A	Shift (Å)	-	-5,3	-7,7	1,3	-
**LMCA1**	A	Shift (Å)	-	-	-	2,0	-
**PMCA2**	N	Shift (Å)	2,2	0,0	-8,8	0,3	-8,8
**SERCA1A**	N	Shift (Å)	9,0	9,1	-12,2	2,0	-20,8
**SERCA2B**	N	Shift (Å)	-	-0,1	-9,8	1,7	-
**SPCA1**	N	Shift (Å)	-	3,4	2,5	2,5	-
**LMCA1**	N	Shift (Å)	-	-	-	3,0	-
**PMCA2**	P	Shift (Å)	-0,1	0,2	1,1	-0,2	0,4
**SERCA1A**	P	Shift (Å)	-0,1	1,8	0,8	0,5	4,0
**SERCA2B**	P	Shift (Å)	-	0,6	-0,1	0,3	-
**SPCA1**	P	Shift (Å)	-	0,7	-1,6	0,8	-
**LMCA1**	P	Shift (Å)	-	-	-	1,5	-
**PMCA2**	A	**Rotation (°)**	15,8	5,8	72,7	21,3	98,3
**SERCA1A**	A	**Rotation (°)**	14,2	27,3	88,4	14,8	93,0
**SERCA2B**	A	**Rotation (°)**	-	14,0	88,9	19,8	-
**SPCA1**	A	**Rotation (°)**	-	19,8	72,3	25,1	-
**LMCA1**	A	**Rotation (°)**	-	-	-	13,8	-
**PMCA2**	N	**Rotation (°)**	8,3	9,9	39,2	7,8	30,6
**SERCA1A**	N	**Rotation (°)**	66,5	84,2	55,1	24,5	28,0
**SERCA2B**	N	**Rotation (°)**	-	23,6	60,7	24,3	-
**SPCA1**	N	**Rotation (°)**	-	79,7	67,2	26,3	-
**LMCA1**	N	**Rotation (°)**	-	-	-	13,2	-
**PMCA2**	P	**Rotation (°)**	4,2	0,5	20,9	8,9	26,0
**SERCA1A**	P	**Rotation (°)**	12,0	11,7	23,2	12,7	36,1
**SERCA2B**	P	**Rotation (°)**	-	10,8	18,6	13,4	-
**SPCA1**	P	**Rotation (°)**	-	15,1	7,9	11,9	-
**LMCA1**	P	**Rotation (°)**	-	-	-	14,3	-

Supplementary Table 2. Cytoplasmic domain movement of PMCA2, SERCA1a, SERCA2b, SPCA1 and LMCA1 between states relevant for this study.

Protein	PMCA2	SERCA1A	SERCA2B	SPCA1	LMCA1
Domain	All	All	All	All	All
E1	2039,60	2287,90	-	-	-
E1-Ca	1902,60	2287,90	3618,50	2248,40	-
E1-Ca-ATP	2161,40	3887,80	4251,10	4283,70	-
E2P	3650,00	4706,30	4889,00	4335,70	4185,00
E2.Pi	2720,20	4156,40	4723,30	4095,80	4216,40
E1-ATP	2161,00	2278,40	-	-	-

Protein	PMCA2	SERCA1A	SERCA2B	SPCA1	LMCA1
Domain	A-N	A-N	A-N	A-N	A-N
E1	0,00	0,00	-	-	-
E1-Ca	0,00	0,00	423,00	x	-
E1-Ca-ATP	0,00	626,30	530,20	565,20	-
E2P	364,30	511,20	437,50	326,90	355,40
E2.Pi	79,30	412,00	368,70	153,50	350,70
E1-ATP	0,00	34,50	-	-	-

Protein	PMCA2	SERCA1A	SERCA2B	SPCA1	LMCA1
Domain	N-P	N-P	N-P	N-P	N-P
E1	512,80	673,90	-	-	-
E1-Ca	532,80	673,90	805,60	552,30	-
E1-Ca-ATP	647,00	840,60	977,90	950,30	-
E2P	458,40	459,60	676,00	483,60	483,40
E2.Pi	483,40	539,70	692,80	554,10	478,20
E1-ATP	615,00	617,90	-	-	-

Protein	PMCA2	SERCA1A	SERCA2B	SPCA1	LMCA1
Domain	P-A	P-A	P-A	P-A	P-A
E1	507,00	470,00	-	-	-
E1-Ca	418,50	470,00	580,60	571,90	-
E1-Ca-ATP	433,70	477,00	617,50	626,40	-
E2P	1002,30	1382,30	1331,00	1357,40	1253,80
E2.Pi	797,40	1126,50	1300,20	1340,30	1279,30
E1-ATP	465,50	486,80	-	-	-

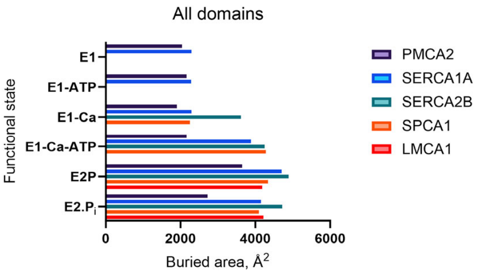

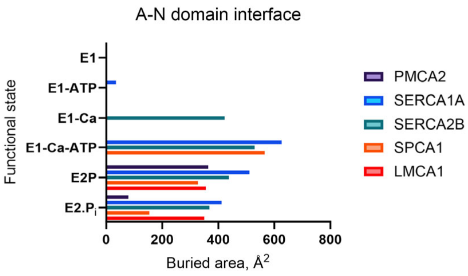

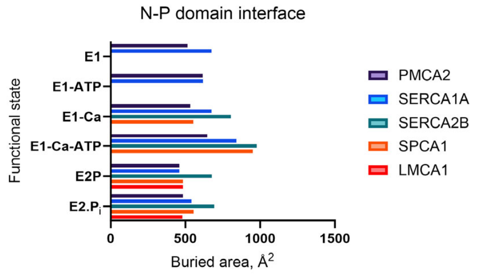

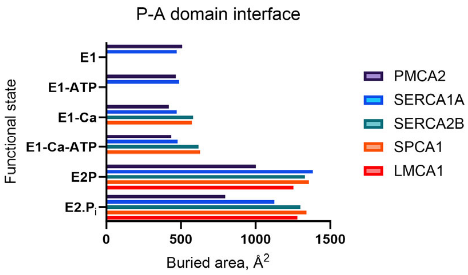

Supplementary Table 3. Extent of cytoplasmic domain interactions of PMCA2, SERCA1a, SERCA2b, SPCA1 and LMCA1 in states relevant for this study. Buried area indicates, in Å^2, the total solvent-accessible surface area of the assembly, buried upon formation of all assemblies' interfaces. For the individual domains, this is divided by 2.

PMCA2

	pKa	
**State**	**D873**	**E412**
E1	6,07	6,69
E1-ATP	6,03	7,73
E1-Ca	11,24	3,82
E1-Ca-ATP	10,97	4,45
E2P	6,9	6,3
E2.Pi	7	5,9

SERCA1a

	pKa	
**State**	**D800**	**E309**
E1	6,88	5,58
E1-ATP	6,48	8,99
E1-Ca	5,09	9,06
E1-Ca-ATP	10,4	6,66
E2P	6,36	6,7
E2.Pi	6,69	6,39

SPCA1

	pKa	
**State**	**D742**	**E308**
~~E1~~		
~~E1-ATP~~		
E1-Ca	6,02	8,13
E1-Ca-ATP	8,86	7,05
E2P	5,95	9,22
E2.Pi	6,81	6,58

Supplementary Table 4. Changes of pKa for Ca^{2+} binding site residues of PMCA2, SERCA1a, and SPCA1 in states relevant for this study.
