# Supplementary material for: Molecular mechanism of ultrafast transport by plasma membrane Ca2+-ATPases
Source: Nature. 2025 Aug 20;646(8083):236–45. doi: 10.1038/s41586-025-09402-3 (PMC12488499; doi:10.1038/s41586-025-09402-3)
Supplement: Supplementary file 2 — Reporting Summary [file 41586_2025_9402_MOESM2_ESM.pdf]

nature portfolio

Corresponding author(s): Stefan Raunser, Bernd Fakler

Last updated by author(s): Jun 17, 2025

Reporting Summary

Nature Portfolio wishes to improve the reproducibility of the work that we publish. This form provides structure for consistency and transparency in reporting. For further information on Nature Portfolio policies, see our Editorial Policies and the Editorial Policy Checklist.

Statistics

For all statistical analyses, confirm that the following items are present in the figure legend, table legend, main text, or Methods section.

n/a	Confirmed	
☒	☐	The exact sample size (*n*) for each experimental group/condition, given as a discrete number and unit of measurement
☒	☐	A statement on whether measurements were taken from distinct samples or whether the same sample was measured repeatedly
☒	☐	The statistical test(s) used AND whether they are one- or two-sided<br>*Only common tests should be described solely by name; describe more complex techniques in the Methods section.*
☒	☐	A description of all covariates tested
☒	☐	A description of any assumptions or corrections, such as tests of normality and adjustment for multiple comparisons
☒	☐	A full description of the statistical parameters including central tendency (e.g. means) or other basic estimates (e.g. regression coefficient) AND variation (e.g. standard deviation) or associated estimates of uncertainty (e.g. confidence intervals)
☒	☐	For null hypothesis testing, the test statistic (e.g. *F*, *t*, *r*) with confidence intervals, effect sizes, degrees of freedom and *P* value noted<br>*Give P values as exact values whenever suitable.*
☒	☐	For Bayesian analysis, information on the choice of priors and Markov chain Monte Carlo settings
☒	☐	For hierarchical and complex designs, identification of the appropriate level for tests and full reporting of outcomes
☒	☐	Estimates of effect sizes (e.g. Cohen's *d*, Pearson's *r*), indicating how they were calculated

Our web collection on statistics for biologists contains articles on many of the points above.

Software and code

Policy information about availability of computer code

Data collection	Cryo-EM data was collected using the commercially available software EPU version 2.8 (ThermoFisher Scientific).
Data analysis	Data collection was monitored live using TranSPHIRE. Intial motion correction and dose weighting was done with MotionCor2 v.1.3.0 and CTF estimation with CTFFIND 4.1.131. SPHIRE software package version 1.4 was used for Particle extraction and 2D classification. Particles were picked automatically with crYOLO version 1.8. 3D classification and refinement was performed with Relion v 3.1 and CryoSPARC v 4.0. Protein model building was carried out in Coot v1.9 and further refined by Phenix v1.18.2. Figures were prepared in Chimera v 1.15 and Chimera X v 1.7.1. X-ray crystallography data processing was carried out in XDS (VERSION Jan 10, 2022 BUILT=20220220) and Phenix v1.18.2 followed by model building and refinement in Coot v1.9 and Phenix v1.18.2, respectively. The residue displacement plot shown in Fig. 2c was generated using a custom script obtained from: https://github.com/schaefer-jh/motionviz.<br><br>msconvert v3.0.11098 was used for processing of primary MS data, MaxQuant 1.6.17 for calibration and quantification of MS data, Mascot 2.7 for database search (UniProtKB/SwissProt release 20181205), and Igor Pro 9 (Wavemetrics) for data fitting and figure preparation.

For manuscripts utilizing custom algorithms or software that are central to the research but not yet described in published literature, software must be made available to editors and reviewers. We strongly encourage code deposition in a community repository (e.g. GitHub). See the Nature Portfolio guidelines for submitting code & software for further information.

Data

Policy information about availability of data

All manuscripts must include a data availability statement. This statement should provide the following information, where applicable:

- Accession codes, unique identifiers, or web links for publicly available datasets
- A description of any restrictions on data availability
- For clinical datasets or third party data, please ensure that the statement adheres to our policy

The cryo-EM maps were deposited in the EMBD with accession ID (dataset in brackets) : EMD-51545 (PMCA-NPTN E1 state), EMD-51546 (PMCA-NPTN E1-ATP state), EMD-51560 (PMCA-NPTN E1-Ca state), EMD-51625 (PMCA-NPTN E1-Ca state used to fit in NPTN Ig1), EMD-51548 (PMCA-NPTN E1-Ca-ATP state), EMD-51544 (PMCA-NPTN E2P state),EMD-51547 (PMCA-NPTN E2.Pi state), EMD-51558 (PMCA alone E2P state) and EMD-51549 (PMCA-alone E1-Ca-ATP state). The atomic coordinates were deposited in the PDB databank under the accession ID (dataset in brackets) : PDB ID 9GSE (PMCA-NPTN E1 state), PDB ID 9GSF (PMCA-NPTN E1-ATP state), PDB ID 9GTB (PMCA-NPTN E1-Ca state), PDB ID 9GSH (PMCA-NPTN E1-Ca-ATP state), PDB ID 9GSD (PMCA-NPTN E2P state),PDB ID 9GSG (PMCA-NPTN E2.Pi state), PDB ID 9GSY (PMCA alone E2P state) and PDB ID 9GSI (PMCA-alone E1-Ca-ATP state),

The atomic coordinates of the mouse NPTN Ig1 domain encompassing residue 29-148 determined by X-ray crystallography were deposited in the PDB databank under the PDB ID 9GTI.

Research involving human participants, their data, or biological material

Policy information about studies with human participants or human data. See also policy information about sex, gender (identity/presentation), and sexual orientation and race, ethnicity and racism.

Reporting on sex and gender	*Use the terms sex (biological attribute) and gender (shaped by social and cultural circumstances) carefully in order to avoid confusing both terms. Indicate if findings apply to only one sex or gender; describe whether sex and gender were considered in study design; whether sex and/or gender was determined based on self-reporting or assigned and methods used.*<br>*Provide in the source data disaggregated sex and gender data, where this information has been collected, and if consent has been obtained for sharing of individual-level data; provide overall numbers in this Reporting Summary. Please state if this information has not been collected.*<br>*Report sex- and gender-based analyses where performed, justify reasons for lack of sex- and gender-based analysis.*
Reporting on race, ethnicity, or other socially relevant groupings	*Please specify the socially constructed or socially relevant categorization variable(s) used in your manuscript and explain why they were used. Please note that such variables should not be used as proxies for other socially constructed/relevant variables (for example, race or ethnicity should not be used as a proxy for socioeconomic status).*<br>*Provide clear definitions of the relevant terms used, how they were provided (by the participants/respondents, the researchers, or third parties), and the method(s) used to classify people into the different categories (e.g. self-report, census or administrative data, social media data, etc.)*<br>*Please provide details about how you controlled for confounding variables in your analyses.*
Population characteristics	*Describe the covariate-relevant population characteristics of the human research participants (e.g. age, genotypic information, past and current diagnosis and treatment categories). If you filled out the behavioural & social sciences study design questions and have nothing to add here, write "See above."*
Recruitment	*Describe how participants were recruited. Outline any potential self-selection bias or other biases that may be present and how these are likely to impact results.*
Ethics oversight	*Identify the organization(s) that approved the study protocol.*

Note that full information on the approval of the study protocol must also be provided in the manuscript.

Field-specific reporting

Please select the one below that is the best fit for your research. If you are not sure, read the appropriate sections before making your selection.

☒ Life sciences ☐ Behavioural & social sciences ☐ Ecological, evolutionary & environmental sciences

For a reference copy of the document with all sections, see nature.com/documents/nr-reporting-summary-flat.pdf

Life sciences study design

All studies must disclose on these points even when the disclosure is negative.

Sample size	Sample size for eight different cryo-EM datasets is given below<br>1. For PMCA-NPTN E1 state 6,676 movies were collected. 292,379 particles were used for final reconstruction.<br>2. For PMCA-NPTN E-Ca state 29,014 movies were collected. 105,000 best particles were used to resolve the neuroplastin terminal domain. 201,573 particles were used to resolve the cytosolic domain and also for final reconstruction.<br>3. For PMCA-NPTN E1-Ca-ATP state 5268 movies were collected. 303,557 particles were used for final reconstruction.<br>4. For PMCA-NPTN E1-ATP state 5788 movies were collected. 185,466 particles were used for final reconstruction.

5. For PMCA-NPTN E2P state 6266 movies were collected. 325,585 particles were used for final reconstruction.
6. For PMCA-NPTN E2.Pi state 4611 movies were collected. 352,232 particles were used for final reconstruction.
7. For PMCA alone E1-Ca-ATP state 8076 movies were collected. 300,272 particles were used for final reconstruction.
8. For PMCA alone E2P state 13134 movies were collected. 473,154 particles were used for final reconstruction.

Data exclusions	During cryo-EM dataset processing, false picks during particle picking were eliminated and further particles that do not contribute to high resolution features have been removed based on 2D and 3D classification which is a standard procedure.
Replication	All cryo-EM dataset were acquired once as it is unattainable to repeat the cryo-EM dataset collection and processing of the same sample from a time and cost perspective.
Randomization	During final cryo-EM dataset reconstruction particles were randomly split into two equal subsets for FSC calculation.
Blinding	Not applicable for this experiment

Reporting for specific materials, systems and methods

We require information from authors about some types of materials, experimental systems and methods used in many studies. Here, indicate whether each material, system or method listed is relevant to your study. If you are not sure if a list item applies to your research, read the appropriate section before selecting a response.

Materials & experimental systems

n/a	Involved in the study	
☒	☐	Antibodies
☐	☒	Eukaryotic cell lines
☒	☐	Palaeontology and archaeology
☒	☐	Animals and other organisms
☒	☐	Clinical data
☒	☐	Dual use research of concern
☒	☐	Plants

Methods

n/a	Involved in the study	
☒	☐	ChIP-seq
☒	☐	Flow cytometry
☒	☐	MRI-based neuroimaging

Eukaryotic cell lines

Policy information about cell lines and Sex and Gender in Research

Cell line source(s)	- tsa201 nptn/basi double knockout (described in Schmidt et al., Neuron, 96: 827-838 (2017), derived from tsa201, Sigma, Cat#96121229)<br>- CHO-K1 (Leibniz Institute DSMZ-German Collection of Microorganisms and Cell Culture, Cat#ACC110)<br>- CHO-K1 at2b2-4 -/-
Authentication	Cell lines were not authenticated
Mycoplasma contamination	There was no contamination
Commonly misidentified lines<br>(See ICLAC register)	Commonly misidentified lines were not used in this study

Plants

Seed stocks	*Report on the source of all seed stocks or other plant material used. If applicable, state the seed stock centre and catalogue number. If plant specimens were collected from the field, describe the collection location, date and sampling procedures.*
Novel plant genotypes	*Describe the methods by which all novel plant genotypes were produced. This includes those generated by transgenic approaches, gene editing, chemical/radiation-based mutagenesis and hybridization. For transgenic lines, describe the transformation method, the number of independent lines analyzed and the generation upon which experiments were performed. For gene-edited lines, describe the editor used, the endogenous sequence targeted for editing, the targeting guide RNA sequence (if applicable) and how the editor was applied.*
Authentication	*Describe any authentication procedures for each seed stock used or novel genotype generated. Describe any experiments used to assess the effect of a mutation and, where applicable, how potential secondary effects (e.g. second site T-DNA insertions, mosiacism, off-target gene editing) were examined.*
